# Does lung function predict the risk of disability pension? An 11-year register-based follow-up study

**DOI:** 10.1186/s12889-020-8277-9

**Published:** 2020-02-03

**Authors:** Irmeli Lindström, Paula Pallasaho, Jouko Remes, Tuula Vasankari, Markku Heliövaara

**Affiliations:** 1 Occupational Medicine, Finnish Institute of Occupational Health, Topeliuksenkatu 41 B, 00250 Helsinki, Finland; 2 Espoo City Health Services, Espoo, Finland; 3 Statistical Services Team, Finnish Institute of Occupational Health, Helsinki, Finland; 4 Finnish Lung Health Association (Filha ry), Helsinki, Finland; 5 Department of Pulmonary Diseases and Clinical Allergology, University of Turku, Turku, Finland; 6 National Institute for Health and Welfare, Helsinki, Finland

**Keywords:** Population-based, Follow-up, Spirometry, Lung disease, COPD, Asthma, Disability

## Abstract

**Background:**

Spirometry is widely used in medical surveillance in occupational health and as a diagnostic test for obstructive and restrictive lung disease. We evaluated the effect of spirometry parameters on the risk of all-cause disability pension in a follow-up study of an occupationally active general population-based cohort.

**Methods:**

We measured the pulmonary function of 3386 currently working participants of the Health 2000 Survey in the clinical phase at baseline using spirometry. We obtained the retirement events of the cohort from the nationwide register for 2000–2011. Cox proportional hazards models were used to determine disability pensions.

**Results:**

At baseline, we identified 111 (3.3%) participants with obstructive spirometry, 95 (2.8%) with restrictive spirometry, and 3180 controls without restriction or obstruction. The age, sex, educational level, body-mass index, co-morbidities (1 or ≥ 2), and the smoking-adjusted hazard ratio of disability pension was 1.07 (95% confidence interval, CI 0.64–1.78) for those with obstructive spirometry, and 1.44 (95% CI 0.89–2.32) for those with restrictive spirometry. As continuous variables, and divided into quartiles, the risk of the lowest quartile of forced ventilation capacity (FVC)% of predicted was 1.49 (95%CI 1.10–2.01) and forced expiratory volume in one second (FEV_1_)% of predicted 1.66 (95%CI: 1.23–2.24) in comparison to the highest quartile in the adjusted models.

**Conclusions:**

Obstructive or restrictive spirometry did not predict disability pension when dichotomized classified variables (normal compared to abnormal) were used. As continuous variables and when divided into quartiles, lower lung volumes showed an increase in the risk of disability pension. Physicians should take this into account when they use spirometry as a prognostic factor of work disability.

## Background

Spirometry is used in medical surveillance in occupational health and as a diagnostic test for obstructive and restrictive lung disease. The most important parameters are forced expiratory volume in one second (FEV_1_) and forced ventilation capacity (FVC). Obstructive impairment is defined as having FEV_1_ in ratio to FVC declined [1, 2]. FVC is declined in restrictive disorders, and FEV_1_ is typically equally declined, thus FEV_1_/FVC remains normal. If both FEV_1_/FVC and FVC are normal, the spirometry is normal, or only slightly abnormal.

Spirometry is the most important diagnostic tool for obstructive lung disease, and FEV_1_/FVC typically declines in prebronchodilator spirometry in obstructive lung diseases. In asthma, in contrast to chronic obstructive pulmonary disease (COPD), lung function may normalize after introducing a bronchodilator or inhaled corticosteroids [3]. The most common cause of mild restriction is obesity. Restriction is typical in lung parenchymal diseases, which are rare in the general population. Sarcoidosis is the most common of these. Spirometry can be normal in milder forms of asthma and lung parenchymal diseases [3]. Thus, the specificity of obstruction or restriction in the prebronchodilator spirometry is low in any lung disease. The sensitivity of prebronchodilator spirometry is high only in COPD, when obstruction is detected [4]. Postbronchodilator spirometry is however always needed to confirm the diagnosis of COPD.

Obstructive lung diseases, asthma and COPD are common health problems among the working-age population and may cause work disability. Follow-up studies have shown that asthma increases the risk of long-term disability [5] and being non-employed [6]. Patel et al. estimated a workforce participation range of 56 to 60% among individuals with COPD and of 65 to 77% among individuals without COPD [7]. Earlier cross-sectional studies have shown that self-reported COPD associates with lower self-reported labour force participation [8–13], while a population-based study showed that spirometry-verified COPD had a minor effect on work ability [14]. Register-based studies [15, 16] have also shown that COPD may affect the frequency and cost of disability. Thornton estimated that COPD associated with a decrease of 8.6% in the likelihood of employment, and an increase of 3.9% in the likelihood of the use of disability insurance [17].

According to The Finnish Centre for Pensions Register, disability pension was granted to 21,304 individuals in 2016, of which only 72 (0.3%) had asthma and 176 (0.8%) had other respiratory disease as their primary diagnosis for disability pension. The most common causes of disability pension are mental disorders and musculoskeletal diseases [18]. Risk factors for disability pension are higher age, low education, being a woman or unmarried [19, 20], physical or psychosocial workload [21], and adverse health behaviour [22–24]. Work-related factors also predict disability pension: for example long work hours, noise exposure, uncomfortable work postures, repetitive or continuous muscle strain, job dissatisfaction, and lack of supervisor support [25].

Our study was carried out among occupationally active people in a nationally representative sample of Finns. Our aim was to find out the impact of spirometry on the risk of all-cause disability. We wanted to know which parameters are the most important predictors of disability and how a physician should interpret spirometry in order to find workers at an increased risk of disability pension and in need of work ability support. We studied whether 1) different spirometric parameters dictomized as normal or abnormal predict disability pension, 2) different spirometric parameters as continous variables predict disability pension, 3) obstruction or restriction is a more important predictor.

## Methods

### Study population

This study was based on the nationally representative Health 2000 Survey, which was carried out in Finland between August 2000 and June 2001 [26, 27]. The population sample of Finnish adults aged 30 or over was formed using a two-stage cluster sampling method [28]. Mainland Finland was divided into five geographical strata based on university hospital districts. In the first stage of sampling, 80 health centre districts (clusters) were selected, and the second stage involved individuals from these districts. The survey had several phases, including many questionnaires, an extensive face-to-face home interview, laboratory and functional capacity tests, and a clinical examination.

We used Vitalograph bellow spirometers (Vitalograph Ltd., Buckingham, UK) to measure lung function. We recorded FEV_1_ and FVC, using the highest readings from at least two technically valid measurements, in accordance with the guidelines [29]. Pulmonary function varies with age, standing height, sex and ethnicity. Therefore, test results need to be compared to the predicted values and lower limits of normal (LLN). We used the global GLI2012 reference values [2] and defined obstruction as having an FEV_1_/FVC below LLN, and restriction as having an FVC below LLN. We used the baseline values, because the bronchodilatation test was performed on only part of the study population.

The severity of airflow obstruction was determined on the basis of FEV_1_% of predicted using the Global Initiative for Chronic Obstructive Lung Disease criteria [4].

A total of 8028 people were sampled, but 51 died before the data was collected. The final sample included 7977 participants, of whom 6986 (88%) were interviewed and 6354 (80%) participated in a health examination [28]. Our study population consisted of those 3386 participants who at the time of baseline examination in 2000–2001 were 1) 30 to 63 years old and 2) full- or part-time employed, and 3) participated in the health examination, including spirometry. We did not include older participants, because the normal retirement age in Finland is 63, and after this it is no longer possible to obtain disability pension. The non-participants in the clinical examinations (*n* = 385) were slightly younger (mean age 42.8 years vs. 44.4 years), more often male (59.2% vs 49.3%), current smokers (40.5% vs 31.5%), and had physician-diagnosed asthma less frequently (6.0% vs 6.6%) than the participants (*n* = 3447).

### Study groups

We first divided the study population into three groups based on the spirometry: the *Obstructive spirometry group* was defined as having an FEV_1_/FVC under LLN in the pre-bronchodilator spirometry. No specific criteria were required for FVC or FEV_1_*.* The *Restrictive spirometry group* was defined as having an FVC under LLN and an FEV_1_/FVC ≥ LLN in the pre-bronchodilator spirometry. The *Controls* were defined as having no obstruction and no restriction in spirometry, i.e. FEV_1_/FVC ≥ LLN and FVC ≥ LLN in pre-bronchodilator spirometry.

We then studied the whole population using the spirometry parameters as continuous variables.

### Disability pensions

The Finnish Centre for Pensions Register provided complete information on all retirement events and their primary and secondary diagnoses granted by the independent pension institutions. All pensions granted before December 31, 2011 were linked to the Health 2000 data by each participant’s personal identification number. The follow-up time of retirement events began when a participant completed the questionnaire and ended when one of the following occurred: 1) retirement due to disability pension, 2) retirement due to other reasons (for example age or unemployment), 3) the end of the follow-up period (December, 312,011), or 4) death.

In Finland, a person with a physician-verified chronic illness, disability, or injury, which has been evaluated as causing considerably reduced work ability, is entitled to a part-time or full-time disability pension [30]. The main outcome of this study was retirement due to disability pension, including permanent, temporary, and part-time disability pensions, as well as ‘individual early retirement pension’, which was available until 2005 for employees born before 1944 who had a long working career and whose work ability was substantially reduced, but who did not fulfil the criteria for disability pension. The primary and secondary diagnosis of disability pension were registered and coded on the basis of the International Classification of Diseases and Related Health Problems 10.

### Covariates

Detailed information on the variables is described elsewhere [28]. The parameters described here are based on the questionnaire data, unless otherwise mentioned.

*Education.* Participants who had completed only comprehensive school or part of high school were classified as having a basic education. Those who had completed vocational school or high-school were classified as having a mid-level education. Those who had completed college or who had some other upper secondary or university degree were classified as having a university-level education.

Asthma was defined as the participant reporting having *doctor-diagnosed asthma* [31]. *Self-reported COPD* was defined as the participant reporting having COPD.

*Smoking.* Participants who had not smoked regularly for at least one year were classified as non-smokers. Ex-smokers had smoked for at least one year and quit at least one month earlier. Participants who currently smoked were classified as current smokers.

*Cotinine* was determined from serum samples collected at baseline and stored at − 20 C°. The method used to determine cotinine concentrations was a modification of the Nicotine Metabolite RIA kit (Diagnostic Products Corporation, LA, USA). For serum cotinine, a high cut-off point of 100 μg was used to separate smokers from non-smokers, as earlier [27].

*Body mass index (BMI)* was based on measured weight and height.

*Other chronic diseases* were defined as having one or more of the following: heart disease (ischemic heart disease/heart insufficiency/heart arrhythmia), stroke, rheumatoid arthritis, chronic low back or neck syndrome, a mental disorder, diabetes, or cancer.

### Statistical analyses

In our preliminary study, we presented the descriptive statistics for participants in three groups as percentages or mean values with standard deviations (SD). After this preliminary study, we fit Cox proportional hazards regression models to the SAS software package (version 9.2; SAS Institute, Inc., Cary, North Carolina). The dependent variable was the first occurrence of any disability pension from 2000 to 2011. Hazard ratios (HR) and confidence intervals (95% CI) were calculated to estimate the effect of the determinants on whether disability pension was awarded, and were adjusted for covariates. We formed a combined variable with the following categories: 1) obstructive spirometry without or with restriction, 2) restrictive spirometry, no obstruction, 3) no obstruction and no restriction in spirometry. We used this categorised variable as an independent variable in the models. The last category was used as a reference category. These analyses consisted of a crude model and five other models using the following independent covariates: 1) age and gender, 2) education and BMI, 3) one comorbidity and two or more comorbidities, 4) all the aforementioned, and 5) all the aforementioned and current or previous smoking and serum cotinine of > 100 μg. We added smoking-related parameters to the model last, because smoking associates closely with obstructive spirometry.

We used Cox proportional hazards regression models with the same adjusting variables as mentioned above, and divided FEV_1_/FVC% of predicted, FEV_1_% of predicted and FVC% of predicted into quartiles (in decreasing order into four groups with an equal number of subjects in each). These groups’ risks of disability pension were compared, using the same adjustments. We finally used Cox proportional hazards regression models with the same adjusting variables, and FEV_1_/FVC% of predicted, FEV_1_% of predicted and FVC% of predicted as continuous variables, divided into quartiles in the risk analysis of disability pension.

## Results

We identified a total of 111 (3.3%) cases with obstructive spirometry and 95 (2.8%) cases with restrictive spirometry at baseline (Table 1). Only one third of participants with obstructive spirometry reported having doctor-diagnosed asthma or COPD. Smoking was common in both the obstructive and restrictive spirometry group. A total of 82% of the participants with restrictive spirometry had a BMI of ≥25.

**Table 1:** Characteristics of study groups at baseline and length of follow-up periods

	Obstructive spirometry *n* = 111	Restrictive spirometry *n* = 95	Controls *n* = 3180	All *n* = 3386
Age,mean years, (SD)	45.9 (7.8)	46.2 (8.4)	44.2 (8.3)	44.3 (8.3)
Males	57 (51%)	60 (63%)	1550 (49%)	1667 (49%)
Education				
Basic	36 (32%)	35 (37%)	686 (22%)	757 (22%)
Mid-level	35 (32%)	41 (43%)	1219 (38%)	1295 (38%)
University	40 (36%)	19 (20%)	1275 (40%)	1334 (39%)
Doctor-diagnosed asthma	25 (23%)	11 (12%)	187 (6%)	223 (7%)
Self-reported COPD	8 (7%)	1 (1%)	10 (0.3%)	19 (1%)
Smoking				
non-smoker	30 (27%)	33 (35%)	1557 (49%)	1620 (48%)
ex-smoker	24 (22%)	25 (26%)	646 (20%)	695 (21%)
current smoker	57 (51%)	37 (39%)	976 (31%)	1070 (32%)
S-cotinine ≥ 100μg/l	59 (53%)	33 (35%)	826 (26%)	918 (27%)
BMI				
< 25	61 (55%)	18 (19%)	1332 (42%)	1411 (42%)
25–29.9	37 (33%)	30 (32%)	1280 (40%)	1347 (40%)
≥ 30	13 (12%)	47 (50%)	567 (18%)	627 (19%)
FEV_1_% predicted				
FEV_1_ ≥ 80%	0 (0%)	70 (74%)	1996 (63%)	2066 (61%)
50 ≤ FEV_1_ < 80	109 (98%)	25 (26%)	1184 (37%)	1318 (39%)
30 ≤ FEV_1_ < 50	2 (2%)	0 (0%)	0 (0%)	2 (0.1%)
FEV_1_ < 30	0 (0%)	0 (0%)	0 (0%)	0 (0%)
Length of follow-up period, mean years (SD)	9.4 (3.0)	8.7 (3.5)	9.7 (2.8)	9.7 (2.8)
Co-morbidities				
1	37 (33%)	29 (31%)	1032 (33%)	1098 (32%)
≥ 2	25 (23%)	24 (25%)	476 (15%)	525 (16%)
Physically active	66 (60%)	45 (48%)	1775 (56%)	1886 (56%)

*FEV1* forced expiratory volume in one second, *FVC* forced ventilation capacity, *BMI* body mass index

During an average 9.7-year follow-up period, 362 (10.6%) participants were granted a disability pension: 16 (14.4%) participants with obstructive spirometry, 19 (20.0%) participants with restrictive spirometry, and 327 (10.3%) of the controls (Table 2). At baseline, these participants were older, slightly more often women, and had a lower level of education, more co-morbidities, and higher BMI than those with no disability pension. The primary or secondary diagnosis of disability pension was a respiratory disease among only 15 (4.1%) of the retired participants, whereas the most common diagnoses were musculoskeletal and psychiatric disorders.

**Table 2:** Proportion of cases (participants with disability pension) in relation to baseline characteristics

	Total n/mean	Cases n/mean	Proportion of cases %	*P*-value
All	3386	362	10.7	
Study groups				
Controls	3180	327	10.3	0.005
Obstructive spirometry	111	16	14.4	
Restrictive spirometry	95	19	20.0	
Background variables				
Age, mean, years	44.3 (8.3)	48.7 (6.6)		< 0.001
Gender				0.018
Male	1667	157	9.4	
Female	1719	205	11.9	
Only basic education	757	127	16.8	< 0.001
Doctor-diagnosed asthma	223	33	14.8	0.040
Self-reported COPD	19	6	31.6	0.012
Chronic brochitis	106	17	16.0	0.055
Comorbidities				< 0.001
0	1763	111	6.3	
1	1098	123	11.2	
≥ 2	525	128	24.4	
Smoking				
Non-smoker	1620	149	9.2	0.026
Ex-smoker	695	83	11.9	
Current smoker	1070	130	12.1	
Cotinine > 100 μg	918	124	13.5	0.001
BMI				< 0.001
< 25	1411	115	8.2	
25–29.9	1347	151	11.2	
≥ 30	627	96	15.3	
Physically active	1886	195	10.3	0.586

*BMI* body mass index, *FEV1* forced expiratory volume in one second

The age, sex, educational level, BMI, and co-morbidities (1 or ≥ 2), self-reported current or previous smoking, and a serum cotinine level of > 100 μg adjusted HR of disability pension was 1.07 (95%CI 0.64–1.78) for obstructive spirometry, and 1.44 (95% CI 0.89–2.32) for restrictive spirometry group in the Cox regression models (Table 3).

**Table 3:** Multivariate models of explanatory variables for subsequent disability pension. Hazard ratios (HR) and confidence intervals (95% CI) are calculated using Cox regression

	Model 1* Age and gender HR (95% CI)	Model 2* Education and BMI HR (95% CI)	Model 3* Co-morbidities 1 and 2 or more HR (95% CI)	Model 4* All mentioned HR (95% CI)	Model 5* All aforementioned, smoking and cotinine HR (95% CI)
Study groups					
Controls	ref	ref	ref	ref	ref
Obstructive spirometry	1.31 (0.79–2.17)	1.41 (0.85–2.34)	1.25 (0.76–2.06)	1.20 (0.72–2.00)	1.07 (0.64–1.78)
Restrictive spirometry	2.00 (1.26–3.18)	1.52 (0.95–2.44)	1.89 (1.19–3.01)	1.50 (0.93–2.41)	1.44 (0.89–2.32)
Age**	1.12 (1.11–1.14)			1.11 (1.09–1.13)	1.11 (1.09–1.13)
Gender					
Men	1.27 (1.03–1.57)			1.26 (1.02–1.56)	1.28 (1.03–1.59)
Women	ref			ref	ref
BMI					
< 25		ref		ref	ref
25–29.9		1.38 (1.08–1.76)		1.17 (0.91–1.50)	1.20 (0.93–1.54)
≥ 30		1.83 (1.39–2.42)		1.28 (0.96–1.70)	1.34 (1.00–1.78)
Education					
High		ref		ref	ref
Mid-level		1.98 (1.51–2.60)		2.07 (1.57–2.72)	1.99 (1.51–2.62)
Basic		2.96 (2.22–3.93)		2.00 (1.50–2.67)	1.86 (1.39–2.50)
Co-morbidities					
No			ref	ref	ref
One			1.84 (1.42–2.38)	1.66 (1.28–2.15)	1.65 (1.27–2.13)
Two or more			4.57 (3.54–5.90)	3.40 (2.62–4.40)	3.40 (2.62–4.41)
Cotinine					
< 100					ref
> =100					1.37 (0.88–2.13)
Smoking					
Non-smoker					ref
Ex-smoker					0.96 (0.73–1.27)
Current-smoker					1.09 (0.69–1.72)

* = adjusted for, ** = a continuous variable, *BMI* body mass index

**Fig. 1:**
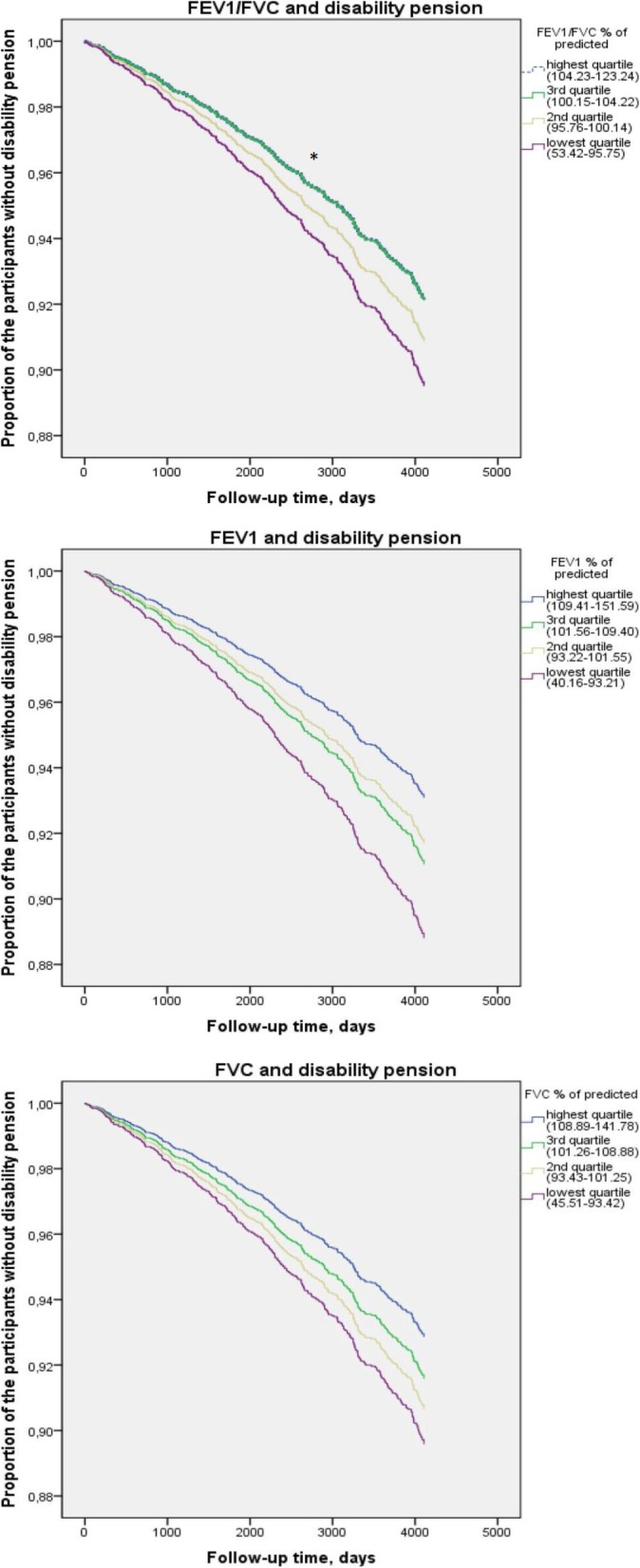
Survival functions for different spirometry parameters and disability pension. The total number of study subjects is 3386 and the number of the participants in each quartile is 846 or 847. Each quartile’s range of the spirometry parameter is in paracenteses. All models are adjusted with age, gender, education level, body mass index, co-morbidities, smoking and cotinine. * In the survival functions of FEV_1_/FVC and disability pension highest and 3^rd^ quartile are equal. FEV_1_= forced expiratory volume in one second, FVC= forced ventilation capacity

When analysing FEV_1_/FVC, FEV_1_ and FVC % of predicted in quartiles with equal number of participants, the risk of disability pension increased gradually as FVC % of predicted decreased (Table 4, Fig. 1). The risk of FVC % of predicted of the lowest quartile was 1.49 (95%CI 1.10–2.01) in comparison to the highest quartile in the models adjusted with the same variables as those used in Table 3. Similarly, FEV_1_% of predicted in the quartiles associated with an increased risk of disability pension: HR 1.66 (95%CI: 1.23–2.24) in the lowest quartile when compared with the highest, whereas the risk of FEV_1_/FVC % of predicted was less clear.

**Table 4:** Multivariate models of the spirometry parameters (% of predicted) and the risk for disability pension. Hazard ratios (HR) and confidence intervals (95% CI) are calculated using Cox regression. The total number of study subjects is 3386 and the number of the participants in each quartile is 846 or 847. Each quartile’s range of the spirometry parameter is in paracenteses

Lung volymes % of predicted (range)	Model 1* Age** and gender HR (95% CI)	Model 2* Education and BMI HR (95% CI)	Model 3* Co-morbidities 1 and 2 or more HR (95% CI)	Model 4* All mentioned HR (95% CI)	Model 5* All aforementioned, smoking and cotinine HR (95% CI)
FEV1/FVC					
highest quartile (104.23–123.24)	ref	ref	ref	ref	ref
3rd quartile (100.15–104.22)	1.07 (0.79–1.45)	1.04 (0.76–1.40)	1.01 (0.74–1.37)	1.03 (0.76–1.40)	1.00 (0.74–1.36)
2nd quartile (95.76–100.14)	1.18 (0.87–1.60)	1.14 (0.84–1.53)	1.08 (0.80–1.45)	1.20 (0.89–1.62)	1.17 (0.86–1.58)
lowest quartile (53.42–95.75)	1.42 (1.06–1.90)	1.41 (1.06–1.89)	1.27 (0.96–1.70)	1.44 (1.07–1.93)	1.35 (1.00–1.83)
FEV1					
highest quartile (109.41–151.59)	ref	ref	ref	ref	ref
3rd quartile (101.56–109.40)	1.29 (0.94–1.78)	1.15 (0.84–1.58)	1.27 (0.92–1.74)	1.31 (0.95–1.80)	1.31 (0.95–1.81)
2nd quartile (93.22–101.55)	1.26 (0.92–1.75)	1.10 (0.79–1.51)	1.18 (0.86–1.63)	1.24 (0.90–1.71)	1.21 (0.87–1.68)
lowest quartile (40.16–93.21)	1.92 (1.43–2.57)	1.63 (1.21–2.19)	1.85 (1.38–2.47)	1.73 (1.28–2.32)	1.66 (1.23–2.24)
FVC					
highest quartile (108.89–141.78)	ref	ref	ref	ref	ref
3rd quartile (101.26–108.88)	1.18 (0.86–1.63)	1.09 (0.79–1.50)	1.21 (0.88–1.67)	1.18 (0.86–1.63)	1.19 (0.86–1.64)
2nd quartile (93.43–101.25)	1.37 (1.00–1.87)	1.22 (0.89–1.67)	1.29 (0.95–1.77)	1.30 (0.95–1.78)	1.33 (0.97–1.82)
lowest quartile (45.51–93.42)	1.66 (1.23–2.23)	1.48 (1.10–2.00)	1.78 (1.33–2.39)	1.50 (1.11–2.03)	1.49 (1.10–2.01)

* = adjusted for, ** = a continuous variable. *FEV1* forced expiratory volume in one second, *FVC* forced ventilation capacity, *BMI* body mass index

As a continuous variable, lower FEV_1_ and FVC % of predicted associated modestly with an increased risk of disability pension (HR 0.98, 95%CI 0.98–0.99 and 0.99, 95%CI 0.98–0.99, respectively in the adjusted models, data not shown in Tables).

## Discussion

In this 11-year register-based follow-up study of the general population, we found no association between obstruction spirometry or restriction and the risk of disability pension when dichotomized classified variables were analysed. Using spirometry parameters as continuous variables and dividing them into quartiles, FVC – a marker of restriction – associated significantly and stepwise increasingly with a risk of disability pension. A relationship was also detected with FEV_1_, whereas the association with FEV_1_/FVC – a marker of obstruction – was less clear. To our knowledge, this is the first study to link spirometry results with a prospective follow up of retirement events from National Registers.

Our main finding is that using dichotomized variables classified as normal or abnormal may cause classification bias and underestimate the importance of lung volumes in predicting work disability by spirometry. Classification bias is typical in cohort studies and was related to the explanatory variable in our study. Using continuous spirometric parameters and dividing them into quartiles is better for identifying individuals at an increased risk of disability. Specially FVC % predicted showed a stepwise increase in the risk of disability when the third, second and lowest quartile were compared to the highest quartile. This refers to a dose-response relationship making FVC % predicted possibly the most important parameter for predicting disability pension. The specificity and sensitivity of the prebronchodilator spirometry result in regard to work limitations is difficult to judge, because it is dependent on the diagnosis of underlining lung disease and its treatment options.

Obstructive spirometry did not associate with the risk of disability pension in our study. The analyses were based on the prebronchodilator test; the cases may partly have reversible obstruction and only some of them true COPD. Obstruction was moderate in 98% of the cases and only one third of those with obstructive spirometry reported having doctor-diagnosed COPD or asthma. Our findings are in line with Finnish statistics on disability pension, and with Erdal et al., who found that productivity losses were minor in a population-based sample of spirometry-verified COPD [14]. The accumulation of more severe COPD cases in earlier studies with self-reported disease [8–13, 17, 32] is possible, because COPD typically has a long silent period with deteriorating lung function but no symptoms and may remain undiagnosed. This might have led to overestimation of the effect of COPD on work disability. Longitudinal studies are rare but have shown that smoking predicts retirement due to COPD [33], and that self-reported COPD increases the risk of dependency on one or more activities of daily living [34].

In the age- and gender-adjusted model, restriction in spirometry associated with a 2.0-fold risk of disability pension in the currently employed general population. Adding education level and BMI to the model reduced this risk to 1.5-fold. Obesity is a common cause of restrictive spirometry, and in earlier studies, it has shown to predict disability pension [22, 35]. Thus, in our study, obesity partly explained the association between restrictive spirometry and disability.

Although we detected no significant relationship between obstruction or restriction as a classified variable in spirometry and disability, we found an association between lower lung volumes as continuous variables and disability. The mechanisms of how low lung volumes affect work ability remain unknown after our study. Low lung function may have an impact on coping in physically demanding work, even if an employee has no lung disease. In previous studies, obstruction in spirometry has been related to all-cause mortality, mortality from cardiovascular and respiratory diseases, and cancer [36].

It is worth noting that the obstructive and restrictive spirometry-associated risk of disability pension changed only slightly when smoking-related variables were added to our models (Table 3). Some studies have shown smoking to be a significant risk factor for disability pension [37, 38]. Haukness et al., however, found that especially among men, this association is mainly explained by the different socioeconomic status of smokers and non-smokers [39]. In our study, only a high serum cotinine level, proving current active smoking, associated with a slightly increased risk of disability pension (Table 3). Self-reported current or previous smoking did not significantly associate with disability pension.

Based on our study, no conclusions can be drawn regarding the prognostic value of spirometry in the risk of disability for individuals with severe lung disease, for example lung fibrosis or severe asthma. Severe lung diseases are rare in the working-age general population, and those suffering from them might have been non-employed at baseline and thus excluded.

The weakness of our study was the lack of postbronchodilator spirometry results. The participants with fixed obstruction may have been at a greater risk of disability pension, which this study was not able to assess. The other weakness was its relatively small number of participants with obstructive and restrictive spirometry. However, the cohort was carefully selected and the participation rate at baseline was as high as 88% for the interview and 80% for clinical examinations. The differences between the participants and the non-participants in the clinical examination were only minor. Thus, we can conclude that our cohort represented the occupationally active general population of Finland in 2000 very well. We took confounding factors into account.

## Conclusions

To conclude, in an occupationally active sample of the general population, obstructive or restrictive spirometry did not predict disability pension when dichotomized classified variables (normal compared to abnormal) were used. When continuous variables were divided into quartiles lower lung volumes showed an increase in the risk of disability pension. Lower lung volumes seemed to have an impact on how people cope in work life. Occupational physicians and other health care providers should take into account declined lung function when, for example, optimizing work tasks, providing care, and considering rehabilitation in order to support work ability.

## Data Availability

The study material is available at: https://thl.fi/fi/tutkimus-ja-kehittaminen/tutkimukset-ja-hankkeet/terveys-2000-2011/tutkimuslomakkeet/terveys-2000-tutkimuksen-suomenkieliset-lomakkeet . The datasets used and analysed during the current study are available from the corresponding author on reasonable request.

## Abbreviations

BMI: Body mass index
CI: Confidence intervals
COPD: Chronic obstructive pulmonary disease
FEV_1_: Forced expiratory volume in one second
FVC: Forced ventilation capacity
HR: Hazard ratios
LLN: Lower limits of normal
